# Identification and Genomic Localization of the *cpe* Gene in *Clostridium perfringens* Strains Associated with Foodborne Outbreaks in South Korea

**DOI:** 10.3390/microorganisms14071399

**Published:** 2026-06-24

**Authors:** Jaehyun Choi, Yeeun Kim, Sumin Ryu, Dabin Kim, Min Jung Lee, Yonghoon Kim, Insun Joo, Woojung Lee

**Affiliations:** Division of Food Microbiology, National Institute of Food and Drug Safety Evaluation, Ministry of Food and Drug Safety, Cheongju 28159, Republic of Korea; wogus58210@korea.kr (J.C.); kye76@korea.kr (Y.K.); banasm@korea.kr (S.R.); dabin29@korea.kr (D.K.); ymin0110@korea.kr (M.J.L.); washout71@korea.kr (Y.K.); jis901@korea.kr (I.J.)

**Keywords:** *Clostridium perfringens*, *cpe* gene, foodborne outbreaks, whole-genome sequencing (WGS), comparative genomics

## Abstract

*Clostridium perfringens* is a major foodborne pathogen in which the genomic localization of the enterotoxin gene, *cpe*, plays an important epidemiological role. In this study, four isolates associated with independent foodborne outbreaks in South Korea were analyzed using complete genome sequencing. All isolates were *cpe*-positive, including three strains carrying chromosomal *cpe* (c-*cpe*) and one strain carrying plasmid-borne *cpe* (p-*cpe*). To provide a broader genomic context, complete genomes retrieved from the National Center for Biotechnology Information database were also analyzed. Most *cpe*-positive strains carried p-*cpe*, whereas c-*cpe* strains were relatively uncommon. Whole-genome analysis revealed a distinct separation between c-*cpe* and p-*cpe* strains based on conserved core-genome features and virulence gene profiles. In c-*cpe* strains, the *cpe* gene was consistently located between the *nadA*–*C* operon and a downstream nucleobase transporter gene and was flanked by IS1470 family transposases, suggesting a conserved chromosomal structure and a possible vertical inheritance. Conversely, p-*cpe* strains carried *cpe* on conserved pCW3-like plasmids, indicating that horizontal gene transfer mediated by a specific plasmid lineage contributes to *cpe* dissemination across diverse genetic backgrounds. Overall, these findings show that *cpe* localization is associated with distinct genomic patterns in *C. perfringens*.

## 1. Introduction

*Clostridium perfringens* is a Gram-positive, spore-forming, rod-shaped anaerobe widely distributed in the intestinal microbiota of humans and animals, as well as in food and environmental sources [1,2]. Although *C. perfringens* commonly exists as a commensal organism, certain strains are important pathogens capable of causing various diseases, including gas gangrene, foodborne gastroenteritis, antibiotic-associated diarrhea, and necrotic enteritis, in humans and animals [1,2,3]. The pathogenicity of *C. perfringens* is primarily mediated by the production of diverse toxins and extracellular enzymes that contribute to tissue destruction and gastrointestinal disease development [4].

*C. perfringens* is currently classified into seven toxinotypes (A–G) based on the presence of six major toxin genes: *plc*/*cpa* (α-toxin), *cpb* (β-toxin), *etx* (ε-toxin), *iap*/*ibp* (ι-toxin), *cpe* (enterotoxin), and *netB* [5]. However, recent genomic analyses have identified atypical strains carrying *cpa*, *cpe*, and *netB* simultaneously, such as the bovine-derived isolate CP280 reported in Japan [6]. These findings suggest that *C. perfringens* continues to diversify genetically through plasmid acquisition and horizontal gene transfer rather than strictly conforming to established toxinotype classifications. Therefore, in addition to refining classification frameworks, continuous genomic surveillance is necessary to monitor emerging variants and assess their epidemiological significance.

From a public health perspective, *C. perfringens* is among the leading causes of foodborne illness worldwide and is responsible for many cases of gastroenteritis each year. In South Korea, the pathogen is consistently detected in foodborne outbreaks and routine surveillance programs, highlighting its continued importance in food safety management [7,8,9].

A key determinant of *C. perfringens* pathogenicity is the enterotoxin gene, *cpe*, which is directly associated with gastrointestinal symptoms. Notably, the genomic location of *cpe* varies among strains, as the gene may be chromosomally encoded or plasmid-borne [10]. This distinction is associated with important epidemiological and phenotypic differences. Previous studies have shown that chromosomal *cpe* (c-*cpe*) strains are commonly associated with typical foodborne outbreaks and exhibit greater environmental resilience, whereas plasmid-borne *cpe* (p-*cpe*) strains are more frequently detected in non-foodborne gastrointestinal disease cases and show greater genomic variability due to plasmid-mediated gene transfer [11,12,13,14].

Recent genomic studies have highlighted evolutionary divergence between c-*cpe* and p-*cpe* lineages, with c-*cpe* strains forming relatively conserved phylogenetic clusters, whereas p-*cpe* strains exhibit greater diversity driven by mobile genetic elements, including conjugative plasmids and accessory virulence factors [15,16,17]. These differences suggest that the genomic location of *cpe* is central to shaping the transmission dynamics, ecological adaptation, and outbreak potential of *C. perfringens*.

However, comprehensive genomic analyses that integrate *cpe* localization, phylogeny, and virulence profiles remain limited, particularly in region-specific settings such as South Korea. Previous studies have primarily focused on specific countries or host populations, and their findings may not be directly applicable to South Korea because of differences in food consumption patterns, antimicrobial use, and environmental conditions. To date, no comprehensive genomic assessment has integrated *cpe* localization, virulence gene profiles, and phylogenetic relationships among outbreak-associated isolates in Korea.

These observations highlight the need for further genomic investigation of *C. perfringens*, particularly regarding differences between c-*cpe* and p-*cpe* strains.

In this study, the genomic characteristics of *C. perfringens* isolates associated with foodborne outbreaks in South Korea were evaluated, with emphasis on *cpe* localization, phylogenetic relationships, and virulence gene profiles.

## 2. Materials and Methods

### 2.1. Bacterial Strains and Genomic DNA Extraction

A total of four *C. perfringens* isolates were obtained from four independent foodborne outbreaks that occurred in South Korea. All isolates were cultured on tryptic soy agar plates and incubated under anaerobic conditions at 37 °C for 24 h using the Anaerogen system (Thermo Scientific, Waltham, MA, USA). Genomic DNA was extracted using a DNeasy Blood & Tissue Kit (Qiagen, Hilden, Germany) according to the manufacturer’s protocol. DNA integrity and concentration were assessed using standard agarose gel electrophoresis and a Qubit^TM^ 3.0 Fluorometer (Life Technologies, Carlsbad, CA, USA), respectively.

### 2.2. Whole-Genome Sequencing and Genome Assembly

Genomic DNA extracted from the four *C. perfringens* strains was subjected to whole-genome sequencing using short- and long-read sequencing platforms. Long-read sequencing was performed on the MinION platform (Oxford Nanopore Technologies, Oxford, UK) using the SQK-NBD114.24 Native Barcoding Kit (Oxford Nanopore Technologies, Oxford, UK). In parallel, short-read sequencing was conducted on the MiSeq platform (Illumina, San Diego, CA, USA) using the MiSeq Reagent Kit v3 (600-cycle). The resulting short-read (Illumina MiSeq) and long-read (Oxford Nanopore Technologies MinION) data were de novo assembled in hybrid mode using Unicycler v0.4.8 [18] with default parameters. Assembly quality was evaluated based on total assembly size, GC content, contig structure, and N50 values. Detailed genomic features are summarized in Table 1.

### 2.3. Genome Annotation and Virulence Profiling

All assembled sequences were annotated using Prokka v1.14.5 [19]. Virulence factors and antimicrobial resistance genes were identified using ABRicate v1.0.1 with multiple databases, including VFDB and ResFinder. Plasmids were identified from hybrid genome assemblies based on circular contig structures and the presence of plasmid-associated genes. Circular contigs containing replication initiation (*rep*) genes and conjugation-related genes were considered putative plasmids.

Plasmid types were classified according to the presence of characteristic marker genes associated with known *C. perfringens* plasmid families [20,21]. Plasmids harboring *pcp* locus genes (e.g., *pcpT* or *pcpS*) were classified as members of the pCP13-like family, whereas those carrying *tcp* conjugation genes (*tcpA*–*tcpJ*) were classified as pCW3-like plasmids [22].

### 2.4. Phylogenetic Analysis

Ninety-four *cpe*-positive genomes retrieved from publicly available complete genomes in the National Center for Biotechnology Information GenBank database were included in the analysis (Table S1). Single-nucleotide polymorphism (SNP) analysis was conducted using the National Genome Information Network for Foodborne Pathogen (NGIN-F) of the Ministry of Food and Drug Safety (https://nginf.nifds.go.kr/cm/main.do; accessed on 9 April 2026), with *C. perfringens* ATCC 13124 used as the reference genome. SNP calling within the NGIN-F pipeline was based on Snippy v4.6.0 (https://github.com/tseemann/snippy; accessed on 9 April 2026) using default parameters. Maximum-likelihood phylogenetic trees were constructed from extracted and dereplicated SNP alignments using IQ-TREE v2.0.7 with the GTR + G + ASC substitution model and 1000 ultrafast bootstrap replicates. Tree annotation was conducted using iTOL v7.2.2 [23].

### 2.5. Comparative Analysis of the cpe-Associated Genomic Region

Genomic regions surrounding the *cpe* gene were extracted from complete genome sequences and compared across isolates. Gene cluster alignment and visualization were conducted using clinker [24], a tool that identifies homologous genes based on sequence similarity and generates comparative plots. The resulting alignments were visualized using clustermap.js, in which gene orientation was indicated by arrows and sequence similarity between homologous regions was represented by shaded connections.

## 3. Results

### 3.1. Genomic Features of C. perfringens Strains

Ninety-eight *C. perfringens* genomes were included in this study. These comprised four isolates obtained from independent foodborne outbreaks in South Korea between 2021 and 2024 and 94 publicly available complete genomes retrieved from GenBank.

The four outbreak-associated isolates had chromosome sizes ranging from 2,855,682 to 3,003,275 base pairs (bp), with GC contents of approximately 28.19–28.28% (Table 1). The number of predicted coding sequences (CDSs) ranged from 2723 to 3190. Each genome contained 87–93 transfer RNA genes and 12–19 ribosomal RNA genes.

Plasmids were detected in three of the four isolates. Among these, one isolate harbored two plasmids, two isolates carried a single plasmid each, and one isolate lacked a plasmid entirely. The detected plasmids ranged from 24,944 to 75,263 bp. Notably, the *cpe*-harboring plasmid identified in this study was classified as a pCW3-like plasmid.

Across the combined dataset, pCW3-like and pCP13-like plasmids were widely distributed among outbreak-associated isolates and reference genomes, indicating that these plasmid families are prevalent in *C. perfringens* populations. Detailed information on chromosomal sequences, plasmid composition, plasmid families, and accession numbers for all strains is provided in Table S1.

Among the outbreak-associated isolates, three were identified as c-*cpe* strains and one as a p-*cpe* strain. Similarly, among the 94 *cpe*-positive reference genomes, 16 were classified as c-*cpe*, whereas 78 were classified as p-*cpe*. All p-*cpe* strains carried *cpe* on a pCW3-like plasmid (Table 1 and Table S1).

### 3.2. Phylogenetic Clustering and Virulence-Associated Features

**Figure 1:**
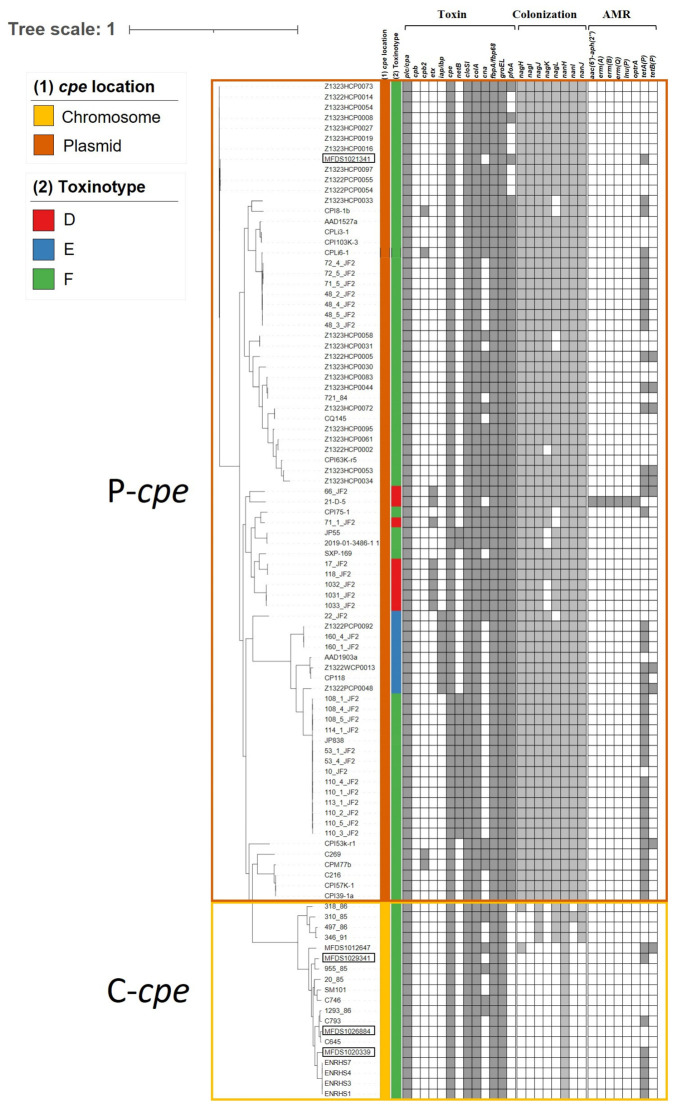
Comparative heatmap of virulence-associated gene profiles according to *cpe* location and toxinotype in *C. perfringens*.

SNP-based phylogenetic analysis revealed clear clustering patterns among the 98 *C. perfringens* isolates (Figure 1). The isolates were broadly separated according to the genomic location of the *cpe* gene, with c-*cpe* (n = 19) and p-*cpe* (n = 79) strains forming distinct phylogenetic groups.

Given that all isolates included in this study were *cpe*-positive, toxinotype distribution was predominantly type F, as expected. Overall, 84 isolates (84/98, 85.7%) were classified as type F, whereas the remaining isolates were classified as type E (8/98, 8.2%) or type D (6/98, 6.1%) (Figure 1).

All isolates carried the core toxin genes *plc*/*cpa*, *colA*, and *cloSI* (98/98, 100%). The pore-forming toxin gene *pfoA* was detected in 92 isolates (92/98, 93.9%). Among the minor toxin genes, *cpb2* was detected in four isolates (4/98, 4.1%), *etx* in eight isolates (8/98, 8.2%), and *netB* in 16 isolates (16/98, 16.3%), whereas the beta-toxin gene *cpb* was not detected in any isolate.

Colonization-associated genes were widely distributed across the dataset (Figure 1). The sialidase gene *nanH* was detected in 96 isolates (96/98, 98.0%), whereas *nanI* and *nanJ* were identified in 78 (78/98, 79.6%) and 80 (80/98, 81.6%) isolates, respectively. Hyaluronidase-associated genes showed variable distribution, with *nagH*, *nagI*, *nagJ*, *nagK*, and *nagL* detected in 74 (75.5%), 72 (73.5%), 73 (74.5%), 65 (66.3%), and 58 (59.2%) isolates, respectively. Notably, p-*cpe* strains carried a broader repertoire of colonization-associated genes, whereas c-*cpe* strains exhibited a comparatively reduced gene repertoire.

AMR genes were infrequently detected across the dataset. The most common genes were *tetA(P)* (42/98, 42.9%) and *tetB(P)* (18/98, 18.4%). In contrast, macrolide resistance genes—such as *erm(A)*, *erm(B)*, and *erm(Q)*, as well as *optrA* and *lnu(P)*—were detected at low frequencies (<5%).

Overall, the combined phylogenetic and genomic analyses revealed that *cpe* localization is strongly associated with distinct genetic backgrounds and functional gene repertoires in *C. perfringens*.

A maximum-likelihood phylogenetic tree based on SNP analysis is shown on the left. The genomic location of the *cpe* gene is indicated by colored bars (yellow, chromosome; orange, plasmid), and toxinotypes are annotated using color codes (red, type D; blue, type E; green, type F). The heatmap shows the presence or absence of virulence-associated genes across all isolates. Dark gray and light gray both indicate gene presence and are used to visually distinguish gene categories, whereas white indicates gene absence. Isolates are ordered according to the phylogenetic tree.

### 3.3. Comparative Analysis of cpe-Associated Genomic Regions

**Figure 2:**
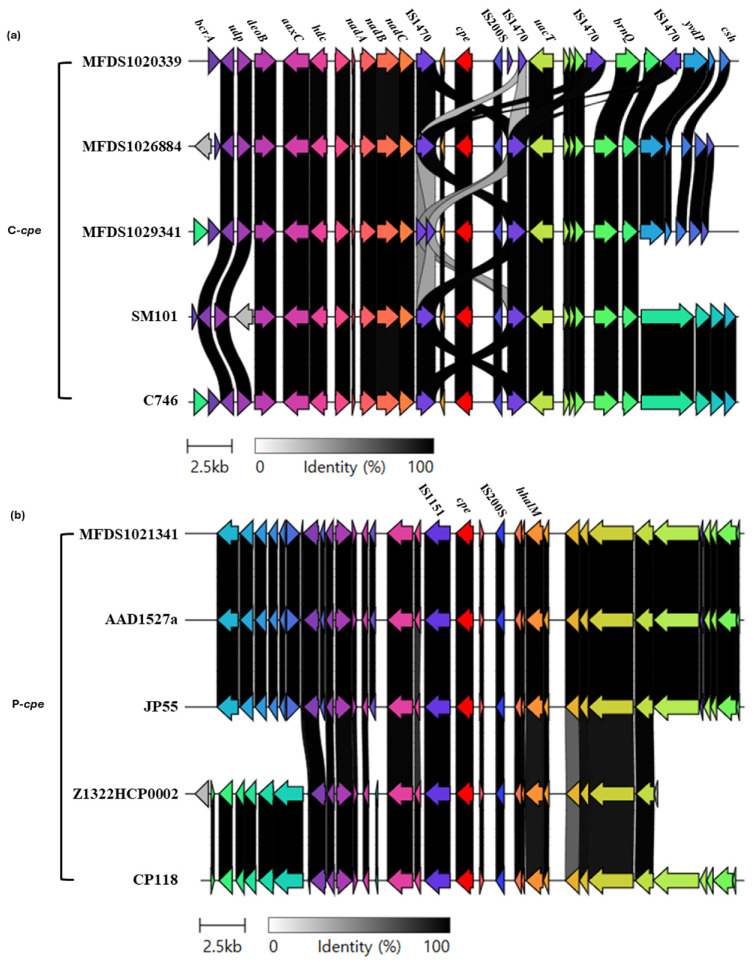
Comparative synteny of *cpe*-associated genomic regions in *C. perfringens.* (**a**) Genomic organization of the chromosomal *cpe* region; (**b**) Genomic organization of the plasmid-borne *cpe* region.

Comparative analysis of genomic regions flanking the *cpe* gene revealed distinct structural differences between the c-*cpe* and p-*cpe* strains (Figure 2).

In c-*cpe* strains, genomic regions flanking the *cpe* locus exhibited highly conserved gene organization across all examined isolates. The *cpe* gene was consistently located between the *nadA–C* operon and the *uacT* gene, indicating a conserved chromosomal insertion site. This region exhibited strong synteny and high sequence identity among c-*cpe* strains and was frequently associated with insertion sequence elements, including IS1470 and IS200.

In contrast, p-*cpe* strains exhibited a distinct plasmid-associated genomic context. Consistent with the findings described above, all p-*cpe* strains carried *cpe* on pCW3-like plasmids, and genomic regions flanking *cpe* were largely conserved across these strains. The *cpe* was inserted at a similar position within the plasmid backbone, although limited structural variation was observed in adjacent regions. Insertion sequence elements, including IS1151 and IS200, were frequently detected near the *cpe* locus together with genes associated with plasmid replication, maintenance, and conjugative transfer.

Overall, these results indicate that c-*cpe* strains possess a highly conserved chromosomal *cpe* insertion site, whereas p-*cpe* strains share a conserved plasmid backbone containing a similarly positioned *cpe* locus with only minor structural variation.

Genomic regions from 10 *C. perfringens* strains (four isolates from this study and six reference strains) were compared using clinker. (a) c-*cpe* regions. (b) p-*cpe* regions. For all strains, genomic regions spanning approximately ±15 kb flanking the *cpe* gene were extracted. Arrows represent predicted CDSs, with arrow direction indicating gene orientation. Homologous regions are connected by shaded links, with shading intensity corresponding to nucleotide sequence identity (0–100%).

## 4. Discussion

In this study, a comprehensive genomic analysis of *C. perfringens* isolates was conducted, with particular emphasis on the genomic location of the enterotoxin gene *cpe*. By integrating outbreak-associated isolates from South Korea with publicly available complete genomes, the analysis revealed that *cpe* localization is strongly associated with phylogenetic structure, virulence gene repertoire, and genomic context. Notably, c-*cpe* and p-*cpe* strains consistently formed distinct genetic groups, indicating that *cpe* localization is not merely a genomic feature but may also contribute to lineage differentiation.

The SNP-based phylogenetic analysis revealed that c-*cpe* strains clustered into a well-defined and genetically coherent lineage, whereas p-*cpe* strains formed a distinct but more dispersed group. However, the broader distribution of p-*cpe* strains within the phylogenetic tree likely reflects differences in genomic architecture, particularly the presence of conjugative plasmids, rather than greater intrinsic genetic diversity. This finding is consistent with previous studies suggesting that c-*cpe* strains represent a conserved evolutionary lineage, whereas p-*cpe* strains are associated with mobile genetic elements that influence genomic organization [25]. The clear separation between these groups further indicates that *cpe* genomic localization is closely associated with the evolutionary structure of *C. perfringens*. Additionally, the tight clustering of c-*cpe* strains indicates a relatively stable genetic background, which may partly explain their frequent association with foodborne outbreaks, as reported previously [7,26].

Differences in virulence-associated gene profiles further support the functional distinction between the c-*cpe* and p-*cpe* strains. Although most isolates shared core toxin genes, clear variation was observed in colonization-associated genes. In particular, comparative genomic analysis demonstrated that p-*cpe* strains harbored a significantly broader repertoire of colonization-associated genes, including the sialidase genes (*nanI* and *nanJ*) and multiple hyaluronidase-associated genes, than c-*cpe* strains (Fisher’s exact test, *p* < 0.001; Table S2). In contrast, c-*cpe* strains exhibited a comparatively reduced repertoire, while *nanH* was conserved in both groups [27,28,29].

Notably, the biological role of *nanH* may extend beyond that of a simple colonization factor. Previous studies have shown that *NanH* is actively produced during sporulation in type F food poisoning strains and enhances the cytotoxic activity of the *cpe* gene product [29]. Given that *cpe* expression is also associated with sporulation, the coexpression of *nanH* and *cpe* suggests a coordinated mechanism that may enhance toxin-mediated pathogenicity. In this context, the consistent presence of *nanH* in c-*cpe* strains, despite their reduced repertoire of additional colonization-associated genes, may represent a streamlined but effective virulence strategy optimized for foodborne infection. In contrast, the broader gene repertoire observed in p-*cpe* strains may reflect greater ecological flexibility and adaptation to diverse host environments.

Comparative analysis of genomic regions flanking the *cpe* gene revealed pronounced structural differences between c-*cpe* and p-*cpe* strains. In c-*cpe* strains, the *cpe* gene was consistently located at a conserved chromosomal insertion site between the *nadA–C* operon and the downstream nucleobase transporter gene, *uacT*. Notably, this genomic region was identified across all analyzed *C. perfringens* chromosomes regardless of *cpe* presence, indicating that it represents a conserved chromosomal backbone. The consistent insertion of *cpe* at this locus suggests that this region may serve as a preferential integration site. Furthermore, IS1470 was consistently observed flanking the *cpe* locus across all examined c-*cpe* strains. The high degree of synteny within this region is consistent with the hypothesis that chromosomal integration of *cpe* may have originated from a conserved insertion event and subsequently been maintained through vertical inheritance within a stable lineage. However, alternative evolutionary scenarios cannot be excluded based on the current data.

In contrast, p-*cpe* strains exhibited a distinct plasmid-associated genomic context. All identified p-*cpe*-associated plasmids belonged to the pCW3-like family, known to be conjugative and capable of mediating interstrain gene transfer [21,30]. Within these plasmids, the *cpe* locus was consistently positioned within a conserved region rather than being randomly distributed, despite being carried on mobile genetic elements. The *cpe* region was also consistently associated with IS1151 in all examined isolates, suggesting that this element may contribute to *cpe* mobilization and dissemination [31]. These findings indicate that horizontal gene transfer mediated by a specific plasmid lineage contributes significantly to shaping the genomic architecture of p-*cpe* strains rather than reflecting unrestricted genomic variability.

The mobility of these plasmids may facilitate the distribution of *cpe* across diverse genetic backgrounds. In contrast, the absence of plasmid-mediated transfer in c-*cpe* strains may contribute to their genomic stability and phylogenetic coherence.

From an epidemiological perspective, our findings provide genomic evidence of a strong association between c-*cpe* strains and foodborne outbreaks [32]. A total of four outbreak-associated isolates collected in South Korea between 2021 and 2024 were available for analysis in this study, which may limit the broader epidemiological interpretation of the findings. Most outbreak-associated isolates analyzed in this study carried chromosomal *cpe*, consistent with previous reports linking c-*cpe* strains to typical food poisoning cases. However, one epidemiologically confirmed outbreak isolate was classified as p-*cpe*.

This observation indicates that although c-*cpe* strains are the predominant lineage associated with foodborne illness, p-*cpe* strains can also cause outbreaks under specific conditions. The conserved genomic background and limited accessory gene variation observed in c-*cpe* strains may be associated with persistence in food-associated environments and outbreak transmission. In contrast, the broader gene repertoire and plasmid-associated genomic architecture of p-*cpe* strains may reflect greater ecological adaptability and the potential for transmission through multiple routes beyond foodborne exposure. These hypotheses were not directly evaluated in the present study, and further investigations are required to clarify the ecological and epidemiological significance of these genomic differences. Therefore, the distinction between c-*cpe* and p-*cpe* strains should not be interpreted as epidemiologically absolute but rather as reflecting differences in predominant transmission patterns and evolutionary strategies.

In conclusion, this study shows that *cpe* genomic localization is closely associated with the evolutionary structure and functional characteristics of *C. perfringens*. Notably, c-*cpe* strains were consistently associated with a conserved chromosomal insertion site within a stable genomic backbone, whereas p-*cpe* strains were linked to a specific conjugative plasmid lineage (the pCW3-like family) that may contribute to *cpe* dissemination. Collectively, these findings highlight the importance of genome-based approaches for understanding the transmission dynamics and evolutionary patterns in this important foodborne pathogen.

## Figures and Tables

**Table 1:** Description of *C. perfringens* strains used in this study.

	MFDS1020339	MFDS1021341	MFDS1026884	MFDS1029341
Isolation year	2021	2022	2023	2024
Chromosome size (bp)	2,861,497	2,855,682	2,933,437	3,003,275
Chromosome GC (%)	28.27	28.19	28.28	28.22
No. of plasmids	-	2	1	1
Plasmid size (bp)	-	75,263; 55,314	24,944	25,540
*cpe* location	Chromosome	Plasmid	Chromosome	Chromosome
tRNAs	92	87	93	91
rRNAs	15	12	19	18
CDS	2723	3190	2761	2896
Accession no.	JBYJEE000000000	JBYJED000000000	JBYJEC000000000	JBYJEB000000000

## Data Availability

The original contributions presented in this study are included in the article. Further inquiries can be directed to the corresponding author.

## Supplementary Materials

The following supporting information can be downloaded at: https://www.mdpi.com/article/10.3390/microorganisms14071399/s1, Table S1: Chromosomal and plasmid profiles. Table S2: Comparison of colonization-associated genes between c-*cpe* and p-*cpe* strains.

## Abbreviations

The following abbreviations are used in this manuscript:

AMR	Antimicrobial resistance
CDSs	Coding sequences
SNP	Single-nucleotide polymorphism
WGS	Whole-genome sequencing
