# Supplementary material for: Identification and Genomic Localization of the cpe Gene in Clostridium perfringens Strains Associated with Foodborne Outbreaks in South Korea
Source: Microorganisms. 2026 Jun 24;14(7):1399. doi: 10.3390/microorganisms14071399 (PMC13414169; doi:10.3390/microorganisms14071399)
Supplement: Supplementary file 1 [file microorganisms-14-01399-s001.zip › TABLE S2.pdf]

TABLE S2. Comparison of colonization-associated genes between c-*cpe* and p-*cpe* strains

Gene category	Gene	c-*cpe* (n=16)	p-*cpe* (n=78)	p-value
Sialidase	*nanI*	1 (6.3%)	78 (100%)	< 0.001
Sialidase	*nanJ*	4 (25.0%)	78 (100%)	< 0.001
Hyaluronidase	*nagH*	2 (12.5%)	78 (100%)	< 0.001
Hyaluronidase	*nagI*	0 (0%)	78 (100%)	< 0.001
Hyaluronidase	*nagJ*	4 (25.0%)	78 (100%)	< 0.001
Hyaluronidase	*nagK*	0 (0%)	72 (92.3%)	< 0.001
Hyaluronidase	*nagL*	0 (0%)	71 (91.0%)	< 0.001
