# Supplementary material for: Identification and Genomic Localization of the cpe Gene in Clostridium perfringens Strains Associated with Foodborne Outbreaks in South Korea
Source: Microorganisms. 2026 Jun 24;14(7):1399. doi: 10.3390/microorganisms14071399 (PMC13414169; doi:10.3390/microorganisms14071399)
Supplement: Supplementary file 1 [file microorganisms-14-01399-s001.zip › TABLE S1.pdf]

TABLE S1. Chromosomal and plasmid profiles

Strain	Chromosome size (bp)	Chromosome Accession No.	Plasmid					
			cpe-harbouring	Size (bp)	Accession No.	Other_1	Size (bp)	Accession No.
SM101	2,897,393	CP000312.1	-	-	-	-	-	-
MFDS1012647	2,954,425	CP106926.1	-	-	-	pCP13-like family	61,476	CP106927.1
20/85	2,927,516	CP075977.1	-	-	-	-	-	-
310/85	2,993,781	CP075968.1	-	-	-	pCP13-like family	57,459	CP075969.1
318/86	2,902,425	CP075966.1	-	-	-	pCP13-like family	56,620	CP075967.1
346/91	3,034,476	CP075964.1	-	-	-	-	-	-
97/86	2,953,417	CP075961.1	-	-	-	pCP13-like family	56,257	CP075962.1
955/85	2,990,614	CP075945.1	-	-	-	-	-	-
1293/86	2,964,677	CP076004.1	-	-	-	pCP13-like family	59,188	CP076005.1
C645	2,962,083	CP075956.1	-	-	-	-	-	-
C746	2,960,095	CP075950.1	-	-	-	pCP13-like family	61,154	CP075951.1
C793	2,967,945	CP075947.1	-	-	-	-	-	-
ENRHS1	3,043,316	CP120689.1	-	-	-	-	-	-
ENRHS3	3,042,868	CP120745.1	-	-	-	-	-	-
ENRHS4	3,014,272	CP120756.1	-	-	-	-	-	-
ENRHS7	3,014,093	CP120747.1	-	-	-	-	-	-
JP55	3,347,300	CP010993.1	pCW3-like family	36,664	CP013042.1	pCP13-like family	42,209	CP013044.1
JP838	3,530,414	CP010994.1	pCW3-like family	48,597	CP013039.1	pCW3-like family	72,750	CP013040.1
CPM 77b	3,180,326	CP075908.1	pCW3-like family	70,476	CP075910.1	pCP13-like family	53,676	CP075909.1
CPLi 6-1	3,358,810	CP075912.1	pCW3-like family	70,476	CP075913.1	pCP13-like family	53,677	CP075914.1
CPLi 3-1	3,294,158	CP075917.1	pCW3-like family	75,262	CP075918.1	pCP13-like family	58,584	CP075919.1
CPI 75-1	3,297,436	CP075922.1	pCW3-like family	75,068	CP075923.1	pCP13-like family	62,433	CP075924.1
CPI 63K-r5	3,360,561	CP075927.1	pCW3-like family	75,258	CP075928.1	pCP13-like family	58,111	CP075929.1
CPI 57K-1	3,149,559	CP075932.1	pCW3-like family	71,757	CP075933.1	-	-	-
CPI 53k-r1	3,274,194	CP075934.1	pCW3-like family	48,590	CP075937.1	pCW3-like family	70,011	CP075936.1
CPI 39-1a	3,137,875	CP075941.1	pCW3-like family	71,757	CP075942.1	-	-	-
721/84	3,264,340	CP075953.1	pCW3-like family	75,262	CP075954.1	-	-	-
C269	3,208,157	CP075970.1	pCW3-like family	70,475	CP075971.1	pCP13-like family	54,958	CP075972.1
C216	3,148,944	CP075973.1	pCW3-like family	71,757	CP075974.1	-	-	-

CPI 18-1b	3,254,784	CP075981.1	pCW3-like family	70,476	CP075982.1	pCP13-like family	53,677	CP075983.1
CPI 103K-3	3,256,086	CP075988.1	pCW3-like family	75,262	CP075989.1	pCP13-like family	58,584	CP075990.1
AAD1903a	3,437,616	CP075992.1	pCW3-like family	67,493; 49,937	CP075993.1; CP075994.1	-	-	-
AAD1527a	3,303,908	CP076000.1	pCW3-like family	75,262	CP076002.1	pCP13-like family	58,584	CP076001.1
CP118	3,390,385	AP025227.1	pCW3-like family	67,488	AP025228.1	-	-	-
21-D-5	3,304,536	CP119188.1	pCW3-like family	121,548	CP119190.1	-	-	-
SXP-169	3,328,661	CP143492.1	pCW3-like family	93,342	CP143493.1	pCP13-like family	30,096	CP143496.1
2019-01-3486-1_1	3,357,566	CP134262.1	pCW3-like family	48,498	CP134266.1	pCW3-like family	72,367	CP134264.1
Z1323HCP0097	3,299,198	CP148588.1	pCW3-like family	75,262	CP148589.1	pCP13-like family	56,015	CP148590.1
Z1323HCP0095	3,270,644	CP148593.1	pCW3-like family	74,003	CP148594.1	pCP13-like family	58,200	CP148595.1
Z1323HCP0083	3,336,339	CP148598.1	pCW3-like family	49,979	CP148600.1	pCP13-like family	58,268	CP148599.1
Z1323HCP0073	3,325,452	CP148601.1	pCW3-like family	75,262	CP148602.1	pCP13-like family	56,016	CP148604.1
Z1323HCP0072	3,352,055	CP148606.1	pCW3-like family	73,382	CP148607.1	pCP13-like family	49,340	CP148609.1
Z1323HCP0061	3,290,419	CP148610.1	pCW3-like family	75,262	CP148611.1	pCP13-like family	58,261	CP148612.1
Z1323HCP0058	3,269,027	CP148613.1	pCW3-like family	75,262	CP148614.1	pCP13-like family	56,015	CP148615.1
Z1323HCP0054	3,338,206	CP148618.1	pCW3-like family	75,262	CP148619.1	pCP13-like family	56,016	CP148620.1
Z1323HCP0053	3,322,565	CP148621.1	pCW3-like family	74,869	CP148622.1	pCP13-like family	49,332	CP148623.1
Z1323HCP0044	3,334,703	CP148624.1	pCW3-like family	74,868	CP148625.1	pCP13-like family	58,269	CP148626.1
Z1323HCP0034	3,269,812	CP148629.1	pCW3-like family	72,982	CP148630.1	pCP13-like family	49,308	CP148632.1
Z1323HCP0033	3,242,572	CP148633.1	pCW3-like family	75,262	CP148634.1	pCP13-like family	52,633	CP148635.1
Z1323HCP0031	3,306,181	CP148638.1	pCW3-like family	75,262	CP148639.1	-	-	-
Z1323HCP0030	3,351,004	CP148641.1	pCW3-like family	75,262	CP148642.1	pCP13-like family	58,448	CP148643.1
Z1323HCP0027	3,324,928	CP148644.1	pCW3-like family	75,262	CP148645.1	pCP13-like family	56,016	CP148646.1
Z1323HCP0019	3,305,770	CP148648.1	pCW3-like family	75,263	CP148649.1	pCP13-like family	56,016	CP148650.1
Z1323HCP0016	3,303,086	CP148652.1	pCW3-like family	75,263	CP148653.1	pCP13-like family	56,016	CP148654.1
Z1323HCP0008	3,329,081	CP148657.1	pCW3-like family	75,262	CP148658.1	pCP13-like family	56,016	CP148659.1
Z1322WCP0013	3,395,553	CP148684.1	pCW3-like family	67,348	CP148685.1	-	-	-
Z1322PCP0092	3,365,298	CP148701.1	pCW3-like family	65,975	CP148702.1	-	-	-
Z1322PCP0055	3,351,386	CP148712.1	pCW3-like family	75,262	CP148713.1	pCP13-like family	56,015	CP148714.1
Z1322PCP0054	3,351,639	CP148716.1	pCW3-like family	75,262	CP148717.1	pCP13-like family	56,015	CP148718.1
Z1322PCP0048	3,428,314	CP148720.1	pCW3-like family	62,976	CP148721.1	pCW3-like family	47,332	CP148722.1
Z1322HCP0014	3,324,930	CP148725.1	pCW3-like family	75,262	CP148726.1	pCW3-like family	56,016	CP148727.1

Z1322HCP0005	3,259,091	CP148729.1	pCW3-like family	76,982	CP148730.1	pCW3-like family	59,719	CP148731.1
Z1322HCP0002	3,274,400	CP148733.1	pCW3-like family	75,262	CP148734.1	pCW3-like family	58,261	CP148735
CQ145	3,291,033	CP159337.1	pCW3-like family	130,020	CP159339.1	-	-	-
110_3_JF2	3,509,000	CP197917.1	pCW3-like family	51,341	CP197919.1	pCW3-like family	72,750	CP197918.1
110_2_JF2	3,509,000	CP197922.1	pCW3-like family	51,341	CP197924.1	pCW3-like family	72,748	CP197923.1
110_4_JF2	3,509,000	CP197910.1	pCW3-like family	51,339	CP197912.1	pCW3-like family	72,750	CP197911.1
108_1_JF2	3,515,065	CP197973.1	pCW3-like family	48,590	CP197976.1	pCW3-like family	87,825	CP197974.1
1031_JF2	3,388,820	CP197817.1	pCW3-like family	98,878	CP197818.1	pCW3-like family	76,985	CP197819.1
10_JF2	3,484,918	CP197830.1	pCW3-like family	48,590	CP197832.1	pCW3-like family	72,749	CP197831.1
1033_JF2	3,388,820	CP197782.1	pCW3-like family	100,714	CP197783.1	pCW3-like family	76,985	CP197784.1
1032_JF2	3,388,827	CP197793.1	pCW3-like family	100,714	CP197794.1	pCW3-like family	66,356	CP197797.1
72_5_JF2	3,314,299	CP198803.1	pCW3-like family	92,495	CP198804.1	-	-	-
66_JF2	3,419,680	CP198724.1	pCW3-like family	99,796	CP198725.1	pCW3-like family	94,662	CP198726.1
71_5_JF2	3,314,299	CP198813.1	pCW3-like family	70,474	CP198814.1	-	-	-
72_4_JF2	3,314,299	CP198809.1	pCW3-like family	70,476	CP198810.1	-	-	-
71_1_JF2	3,464,641	CP198819.1	pCW3-like family	101,944	CP198821.1	pCW3-like family	103,224	CP198820.1
53_4_JF2	3,528,889	CP198706.1	pCW3-like family	48,591	CP198709.1	pCW3-like family	72,750	CP198707.1
48_5_JF2	3,261,238	CP198518.1	pCW3-like family	70,476	CP198520.1	-	-	-
48_4_JF2	3,275,045	CP198525.1	pCW3-like family	70,476	CP198527.1	-	-	-
22_JF2	3,432,079	CP198450.1	pCW3-like family	134,293	CP198451.1	pCW3-like family	95,728	CP198452.1
17_JF2	3,456,989	CP198502.1	pCW3-like family	84,534	CP198504.1	pCW3-like family	89,505	CP198503.1
48_2_JF2	3,261,238	CP198539.1	pCW3-like family	70,476	CP198541.1	-	-	-
53_1_JF2	3,528,876	CP198714.1	pCW3-like family	48,591	CP198717.1	pCW3-like family	72,750	CP198715.1
48_3_JF2	3,261,238	CP198532.1	pCW3-like family	70,476	CP198534.1	-	-	-
160_1_JF2	3,401,937	CP198215.1	pCW3-like family	65,975	CP198216.1	-	-	-
160_4_JF2	3,401,937	CP198513.1	pCW3-like family	65,975	CP198514.1	-	-	-
114_1_JF2	3,524,769	CP197857.1	pCW3-like family	48,590	CP197860.1	pCW3-like family	72,750	CP197858.1
110_5_JF2	3,509,000	CP197904.1	pCW3-like family	51,341	CP197906.1	pCW3-like family	72,750	CP197905.1
110_1_JF2	3,509,000	CP197928.1	pCW3-like family	51,341	CP197930.1	pCW3-like family	72,750	CP197929.1
108_4_JF2	3,515,064	CP197965.1	pCW3-like family	48,590	CP197968.1	pCW3-like family	72,750	CP197966.1
108_5_JF2	3,515,064	CP197956.1	pCW3-like family	48,589	CP197959.1	pCW3-like family	87,939	CP197957.1
118_JF2	3,458,240	CP198065.1	pCW3-like family	84,534	CP198067.1	pCW3-like family	90,690	CP198066.1

113_1_JF2	3,509,000	CP197869.1	pCW3-like family	51,341	CP197871.1	pCW3-like family	72,750	CP197870.1
